# Therapeutic effect of intraductal saline irrigation in chronic obstructive sialadenitis

**DOI:** 10.1186/s12903-020-01078-7

**Published:** 2020-03-24

**Authors:** Jo-Eun Kim, Sam-Sun Lee, Chena Lee, Kyung-Hoe Huh, Won-Jin Yi, Min-Suk Heo, Soon-Chul Choi

**Affiliations:** 1 Department of Oral and Maxillofacial Radiology, Seoul National University Dental Hospital, Seoul, Republic of Korea; 2 Department of Oral and Maxillofacial Radiology and Dental Research Institute, School of Dentistry, Seoul National University, Seoul, Republic of Korea; 3 Department of Oral and Maxillofacial Radiology, Yonsei University College of Dentistry, Seoul, Republic of Korea

**Keywords:** Sialadenitis, Salivary gland disease, Irrigation, Minimally invasive

## Abstract

**Background:**

The aim of the present study was to evaluate the effectiveness of intraductal irrigation using normal saline in chronic obstructive sialadenitis.

**Methods:**

Patients who had one of the following symptoms were recruited: pain, swelling, stiffness, and dry mouth. A total of 58 salivary glands in 33 patients were diagnosed as having sialadenitis using sialography and ultrasonography. The patients were divided into two groups (swelling group and dry mouth group), according to the major complaint. Repeated intraductal irrigation was performed on each gland. Difference of symptom severity evaluated using numerical rating scale (NRS), and ductal width measured using ultrasonography were compared between the two groups.

**Results:**

The average NRS score was significantly decreased from 6.0 to 3.3 after 3–5 visits of intraductal irrigation (*P* < 0.05). The reduction in NRS was greater in the swelling group than in the dry mouth group, although the difference between the groups was not statistically significant. There was no change of ductal width before and after the irrigation.

**Conclusions:**

Intraductal irrigation according to this study method using normal saline is a simple treatment for the patients with chronic obstructive sialadenitis. It provides a conservative treatment option reducing the subjective symptoms.

## Background

Salivary gland disorders are classified as neoplasms and inflammatory disease; however, the classification of inflammatory disease is not well defined. Individuals with sialadenitis caused by viral or bacterial infections have acute symptoms and are cured by antiviral agents or antibiotics with supportive care. Chronic obstructive sialadenitis (COS), also known as chronic obstructive, recurrent, or chronic sialadenitis, is one of the frequent diseases of the salivary glands and is characterized by recurrent swelling and pain caused by pressure [1]. The representative symptom is swelling related to food intake, called “mealtime syndrome”. A study reported that the incidence of admission for sialadenitis was 27.5 per million of the population [2], and the other report showed that the obstructive sialadenitis accounts for approximately one-half of benign salivary gland diseases [1].

Through the experimental and clinical researches, paradigm has been shifted in the understanding the etiology of COS [3]. Sialomicrolith, which can be made from the stagnated calcium-rich secretory material with phospholipid from damaged cellular membrane, revealed as a causative of obstructive sialadenitis [4]. Other factors which make secretory inactivity, or stagnation of saliva, such as kink of duct, malfunction of ductal muscles, or inflammation causes plugs, also made obstructive sialadenitis [5]. Focal obstructive adenitis (FOA) in the parenchyma has been associated with the impaction of sialomicrolith in small ducts, and inflammation flared with the proliferation of ascending microbes in FOA during periods of secretory inactivity. These vicious cycles resulted in chronic obstructive sialadenitis, and sialoliths can develop secondarily [3, 6].

The conventional treatments for COS were general antibiotics or corticosteroid. When COS patients complained of swelling, palliative treatments like gland massage, hyperhydration, and use of sialagogues were prescribed to relive the discomfort. As the results of experimental studies on etiology have been reported, the demand for more fundamental treatments has increased [7]. Although sialendoscopy is recognized as a new and effective treatment method, it is not available in all countries or medical centers. Also, the use of sialendoscopy is quite limited for its invasiveness due to the diameter of the endoscope which requires delicate skills from physicians [8, 9].. Thus the conservative and non-invasive simple ductal irrigation is necessary for the treatment of COS. Previous studies have shown that they are trying to see drug effects through the method of irrigation using corticosteroid and antibiotics directly within the duct [10, 11]. However, only small number of studies had carried out and the effect between the drug use and irrigation itself has yet been distinguished. Therefore, the aim of the this study was to evaluate the therapeutic effect of intraductal irrigation using normal saline.

## Methods

### Patients

**Fig. 1:**
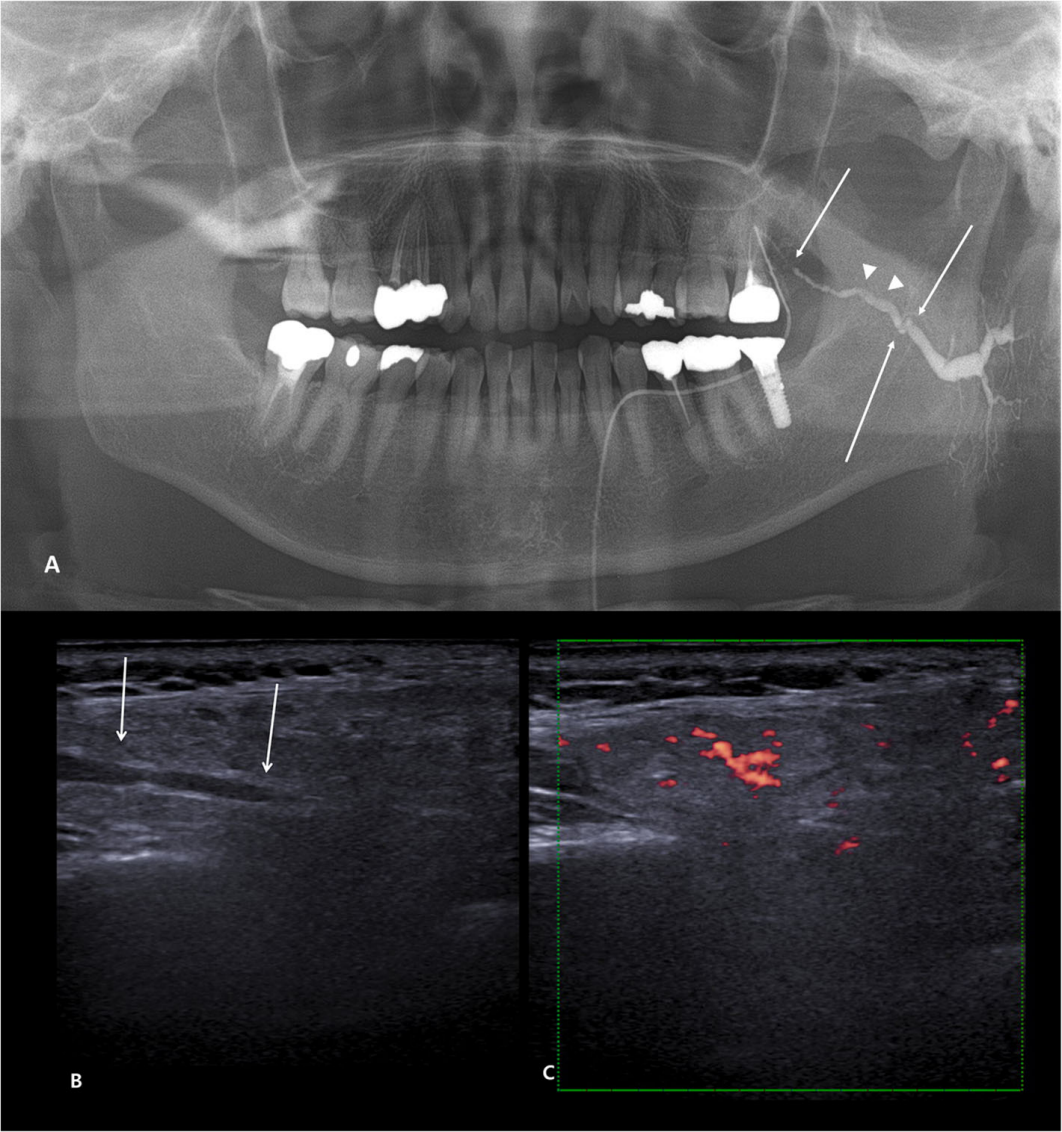
Representative sialography and ultrasonography of patients diagnosed with sialadenitis. **a** Left Stensen’s duct and intraglandular ductile show dilation with stenosis (arrows). Note the irregular surface of ductal filling (arrowheads) suggesting of plugs and coagulated substances. **b** Dilated duct is shown on ultrasonography of the same patient (arrow). **c** Power Doppler image shows increased vascularity, implicating inflammatory state of parenchyma

The Institutional Review Board of Seoul National University Dental Hospital have approved the present study (IRB142/10–18). Patients with at least one of the following subjective symptoms were recruited: pain, swelling, stiffness (particularly when chewing), and dry mouth. On the first visit, sialography using panoramic radiography (OP-100, Instrumentarium Dental, Tuusula, Finland) and ultrasonography (Accuvix A30, Samsung Health Care, Seoul, Korea) were performed by one radiologist (JK). Patients who had ductal dilatation, stenosis, and parenchymal filling (globular or punctate filling) were diagnosed with sialadenitis by sialography (Fig. 1a). Ultrasound imaging features of severe ductal dilatation, increased vascularity and hypoechogenic foci of parenchyma helped diagnosis of COS (Fig. 1b and c). The final diagnosis of sialadenitis was established through the consensus of two radiologists, who majored in oral and maxillofacial radiology and have more than 10 years of experience, on the basis of clinical history and an examination that included sialography and ultrasonography [12, 13]. The measurement of width of duct was measured in the widest portion, and the average value was recorded by the two radiologists.

The patients who had sialolith, tumor, severe acute inflammation leading to pus, and/or history of medication/hospitalization were excluded.

Patients were classified into two groups following their major complaint; swelling or pain (swelling group) and dry mouth (dry mouth group).

### Intraductal irrigation

The specific method used for intraductal irrigation was as described in the previous technical report [14].

Step 1 (orifice exploration): Following drying of the orifice region, the salivary glands were massaged to identify the orifice. The orifice of the salivary gland was explored using a periodontal probe.

Step 2 (duct dilation): The duct was gradually enlarged using a lacrimal probe (Bowman Lacrymal Probe, Josef Heiss Medical GmbH, Tuttilngen) ranging between #0000 and #0.

Step 3 (saline filling): The duct was cannulated with a scalp vein set (23Gx13/4 in) (JMS Co.Ltd., Tokyo, Japan) following removal of the needle tip, which was blunted with dental Kelly forceps. Normal saline was slowly introduced into the duct and the gland through a cannula using a 5-mL syringe. Saline infusion was terminated when the patients experienced stiffness in the salivary gland, and the approximate volumes were 1.2–1.7 in the parotid gland and 0.7–1.5 in the submandibular gland. Following the infusion of saline, the orifice was plugged and maintained for 5 min.

Step 4 (evacuation): Following removal of the scalp vein set, the gland region was massaged gently to discharge the saline.

The above procedures of step 1 to 4 were repeated three times in every single visit, and all irrigation procedures were performed by one radiologist (JK).

### Evaluation of the effect

On the initial visit, the subjective symptoms and duration of symptoms were recorded, and the degree of discomforts was recorded using a numerical rating scale (NRS) to evaluate therapy. The objective changes were investigated using ultrasonography, which revealed the condition of the gland and duct. Width of dilated duct was measured in the ultrasound image in which the probe posited transversely.

The number (more than 3 times) and the interval (2 or 4 weeks) of visit for irrigations were determined by the clinical symptoms and consequently by the therapeutic responses. Patients with recurrent swelling got irrigation for every 2 weeks and adjusted to every 4 weeks once after the swelling has gone down.

### Data analysis

The difference in NRS, and ductal width between the initial- and the last-irrigation time points were analyzed using the Wilcoxon singed rank test, due to a lack of normality of the data. The changes in NRS and the number of visits between the two groups were compared using a Mann-Whitney test.

**Fig. 2:**
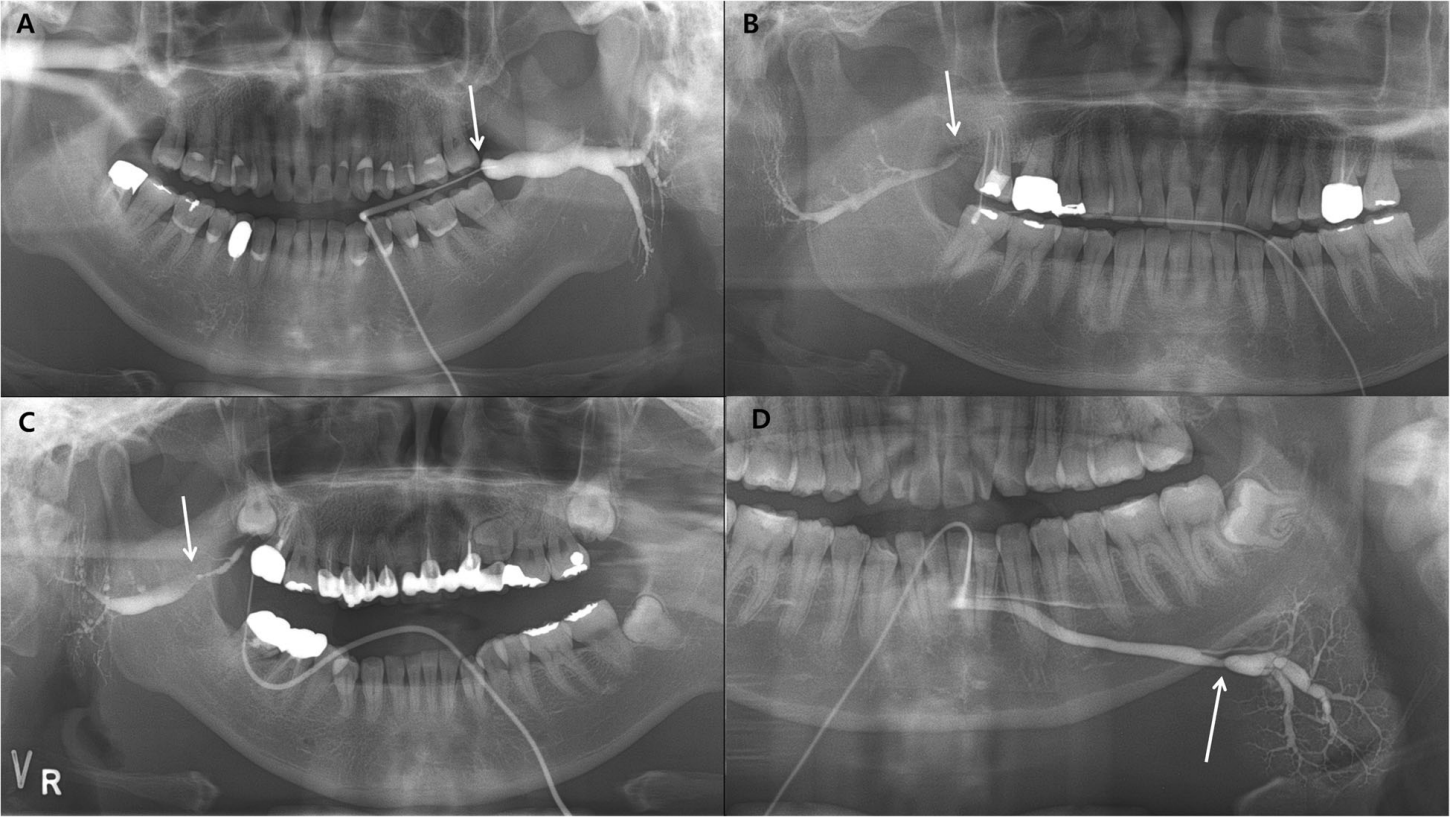
Each images shows classification according to location of major stenosis of salivary duct on sialography. **a** Orifice, **b** distal duct, **c** middle duct, **d** proximal duct

The major stenosis was defined as the largest difference between dilatation and stenosis of duct, when the significant stenosis was shown on sialography in the swelling group (*n* = 19). The duct between orifice and parenchyma was divided into three parts; distal, middle, and proximal, and the location of it was recorded as the orifice, distal duct, middle duct, and proximal duct (Fig. 2). The differences in NRS according to the major stenosis were compared by Kruskal-Wallis test. All statistical analyses were performed using SPSS 23 (IBM Co., Chicago, IL. US).

## Results

A total of 58 salivary glands (39 parotid glands and 19 submandibular glands) from 33 patients were included in the present study, which were diagnosed as sialadenitis using sialography and ultrasonography. The mean age was 52 years (21~83 years) and female predominance was significant (male: female = 5:28). The duration of symptoms varied between 3 days and > 10 years. The most common symptom was swelling (60.6%), followed by dry mouth (39.4%), pain (12.1%), stiffness (9.1%), salty saliva (6.1%), and itching (6.1%). The sialographic findings were ducal dilatation (93.9%), ductal stenosis (63.6%), and acinar filling (42.4%). On ultrasound imaging, dilated ducts were detected in 22 patients, and the average width was 1.63 mm.

The average NRS was 6.0 on the initial visit, and this score decreased to 3.3 on their final visit (Table 1). The average number of visits per patients was 4.1, and the NRS decreased significantly following repeated irrigation (*P* = 0.000). However, the width of the dilated duct remained unchanged on their final visit, with an average width of 1.62 mm (*p* = 0.905).

**Table 1:** Mean numeric rating scale (NRS) of discomfort and ductal width on ultrasonography (US)

	Initial	Last	Difference
N R S			
Total (*n* = 33)	6.0 ± 2.5*	3.3 ± 2.6*	2.7 ± 2.2
Swelling (*n* = 20)	5.9 ± 2.4	2.7 ± 2.5	3.2 ± 2.4
Dry mouth (*n* = 13)	6.1 ± 2.2	4.2 ± 2.4	1.9 ± 1.3
Width of duct on US (mm)			
Total (*n* = 33)	1.63 ± 1.65	1.62 ± 1.64	0.00 ± 0.09
Swelling (*n* = 20)	2.39 ± 1.67	2.38 ± 1.65	0.00 ± 0.12
Dry mouth (*n* = 13)	0.51 ± 0.74	0.52 ± 0.75	0.01 ± 0.02

*The effect of irrigation was statistically significant (*p* < 0.05) by Wilcoxon singed rank test

The average number of visits for irrigation was 4.2 in the swelling group and 3.9 in the dry mouth group, and there was no statistical difference between two groups. The reduction in NRS was greater in the swelling group than in the dry mouth group; the differences were 3.2 and 1.9, respectively, however the difference between the two groups was not statistically significant (*P* = 0.087). According to major symptoms in the swelling and dry mouth groups, the width of duct was significantly wider in the swelling group (2.39 mm) than in the dry mouth group (0.51 mm). There was no significant difference statistically in the decrease of NRS according to the location of stenosis in the duct (Table 2).

**Table 2:** Mean numeric rating scale (NRS) changes according to the location of major stenosis

Location of stenosis	∆ NRS (mean ± sd)
Orifice (*n* = 2)	2.25 ± 2.75
Distal Duct (*n* = 5)	3.60 ± 2.27
Middle Duct (*n* = 6)	4.83 ± 1.84
Proximal Duct (*n* = 6)	2.25 ± 1.15

∆ NRS = Last NRS–Initial NRS

Kruskal-Wallis test was performed. *P* = 0.203

## Discussion

In the present study, the subjective symptoms of patients with chronic sialadenitis were significantly relieved from NRS 6.0 to 3.3 following repeated intraductal irrigations. Few previous articles reported that the effect of salivary gland irrigation [7, 10, 11, 15] and irrigation was good for relieving the COS symptom similar to this study. Most of them were used antibiotics and steroid for irrigation solution. To evaluate the therapeutic effect of irrigation itself, not by the drug effect, two studies comparing the effect of irrigation between using normal saline and other solution were carried out [7, 15]. They reported that the irrigation itself was more important. Whatever the irrigation agents used, the removal of inflammatory substances was a key. Therefore, intraductal irrigation using normal saline may be the most effective conservative treatment without drug side effects.

The main mechanism of intraductal irrigation to COS is the removal of obstructive factors, including microliths, coagulated substances that can act as a nidus for calcification, and inflammatory substances. During the instillation and evacuation of saline, the dilution and flushing of ascending microbes, coagulated proteins, and small sialoliths were performed through the dilated ducts [7]. It also has been reported that the patients who underwent a sialography experienced the unintended benefits of pain reduction [16], and it could be explained by the irrigation effect.

Though it was not statistically significant, the reduction of NRS was marked in the swelling group more than dry mouth group. The symptom of swelling occurs when saliva is produced, because the ductal stenosis and dilatation are formed in the duct. The structural changes like stenosis and dilatation has made the saliva stagnate inside the duct and has triggered the inflammation. This causes more structural changes and turns into a vicious cycle [7, 17]. The structural changes like stenosis and dilatation has made the saliva stagnate inside the mouth and has triggered the inflammation. This causes more structural changes and turns into a vicious cycle. The swelling group also expected that there would be a difference in irrigation effect depending on the location of the major stenosis, because there would be a difference in instrument access depending on the location of stenosis. However, there was no difference, probably because the number of samples was too small. In the dry mouth group, the response to irrigation treatment was less than the swelling group. Patients who complained of dryness were also selected for this study as being diagnosed with sialadenitis by conducting a sialography. However, the symptom of dryness seems to be related to the function of minor salivary glands, which have ongoing spontaneous secretion [3].

Although the reduction of NRS was apparent following repeated irrigation in the present study, the width of the dilated duct remained unchanged. The reason for this is that, once the structural change (such as fibrosis of the duct) occurs, the reversal of this change does not appear to be possible without the surgical procedure [18]. Therefore, the early detection of obstructive symptoms and removal of inflammatory foci is important to prevent permanent structural ductal change.

For the treatment of obstructive sialadenitis, sialendoscopy has been recommended in recent decades. With sialendoscopy, intraductal irrigation and the dilatation of stenosis can be performed under the direct vision. However, there are several practical problems with using sialendoscopy. First of all, the width of the orifice and duct should be > 1.2–1.3 mm wide for the endoscope to be inserted safely [19], and a wider channel is required for the use of working instruments, including the basket and balloon [20]. The size of the orifice of normal salivary glands may be < 1.2 mm. Furthermore, the width of ductal stenosis may be < 1.2 mm in patients with COS. Therefore, the introduction of sialendoscopy requires surgical widening of the orifice [9]. In addition, the endoscopic procedures are strongly dependent on the surgeon’s skill. Complications reported during the endoscopic procedure include avulsion of the duct, minor ductal tears, and superficial mucosal necrosis [9]. One of the adverse effects is the increased cost of the procedure due to the high cost of the equipment. Several reports have shown the effectiveness of salivary gland irrigation using sialendoscopy [8, 21]. However, the use of sialendoscopy only for irrigation is not recommended due to the possibility of additional injury and economic harm as mentioned above. Intraductal irrigation is a relatively simple and easy way to reduce the patients discomfort of chronic obstructive sialadenitis.

## Conclusions

Intraductal irrigation using normal saline is a simple and effective treatment for the patients with chronic obstructive sialadenitis with the symptom of swelling.

## Data Availability

The dataset used and analyzed in the current study are available from the first author on reasonable request (noel1st@snu.ac.kr).

## Abbreviations

COS: Chronic obstructive sialadenitis
NRS: Numerical rating scale
